# Kala-azar Control, Uganda

**DOI:** 10.3201/eid1303.060706

**Published:** 2007-03

**Authors:** Jan H. Kolaczinski, Dagemlidet Tesfaye Worku, François Chappuis, Richard Reithinger, Narcis Kabatereine, Ambrose Onapa, Simon Brooker

**Affiliations:** * Malaria Consortium Africa, Kampala, Uganda; † London School of Hygiene & Tropical Medicine, London, United Kingdom; ‡ Médecins Sans Frontières, Kampala, Uganda; § Médecins Sans Frontières, Geneva, Switzerland; ¶ Geneva University Hospital, Geneva, Switzerland; # Ministry of Health, Kampala, Uganda

**Keywords:** Leishmaniasis, *Leishmania donovani*, visceral, kala-azar, control, Uganda, letter

**To the Editor:** Much of the leishmaniasis in Africa is concentrated in East Africa. In this region, visceral leishmaniasis (kala-azar) is caused by *Leishmania donovani* and is endemic in remote parts of Somalia, Sudan, Ethiopia, Kenya, and Uganda (*1*).

In Uganda, kala-azar is transmitted by the sandfly *Phlebotomus martini,* and transmission is thought to be anthroponotic. Studies in Sudan and Kenya have detected *L. donovani* in domestic animals (*2*,*3*), but whether these play a role in Uganda is unknown. In Uganda, the disease appears to be restricted to Pokot County, a semiarid lowland area in Nakapiripirit District (Appendix Figure). This focus is an extension of a larger focus in West Pokot District in Kenya (*4*). The area is mainly inhabited by the Pokot, a seminomadic tribe of pastoralists. Nakapiripirit is one of the most underserved districts of Uganda, plagued by tribal clashes.

Though kala-azar has been reported in East Africa since the early 1900s, it was not described in Uganda until the 1950s (*5*) and remained largely unnoticed until 1997, when Médecins Sans Frontières (MSF, Swiss Section) began to provide assistance to Amudat Health Centre in Pokot County. In 2000, MSF initiated a kala-azar control program, focusing on passive case detection and treatment.

From January 2000 to February 2006, a total of 3,645 patients suspected of having kala-azar were screened at Amudat Health Centre by using the direct agglutination test or rK39 antigen–based dipsticks (*6*); 2,088 patients with confirmed disease were treated with daily intramuscular injections of sodium stibogluconate or meglumine antimonite, 20 mg/kg bodyweight, for 30 days. Overall, 80% of the patients were <15 years of age, 75% were male, and 70% were from Kenya. From 2000 through 2005, the number of patients treated more than tripled, from 175 to 690 cases per year. Although this increase likely results, at least in part, from greater case detection due to the availability of treatment, we cannot exclude a real increase in disease because kala-azar prevalence in the area is unknown.

Information on local vector behavior and risk factors for infection or disease (e.g., malnutrition and HIV coinfection) is limited, and which potential interventions are appropriate is unclear. A pilot entomologic study in 2004 (J. Stevenson, master’s thesis) demonstrated that termite mounds (Figure) are important vector breeding and resting sites and that the practice of sitting on termite mounds while guarding livestock might increase the risk for infection. In contrast, humans may be protected from kala-azar when in close proximity to livestock (i.e., because of diversion of sandflies to alternative hosts) or when lighting fires indoors (smoke acts as a repellant to most biting flies). Ownership of insecticide-treated nets, which could protect persons from sandfly bites and reduce kala-azar transmission (*7*), was low. Although most of the local population had heard of kala-azar, known locally as *termes*, and regarded it as potentially fatal, few were aware of how kala-azar is transmitted (J. Stevenson, master’s thesis).

**Figure:**
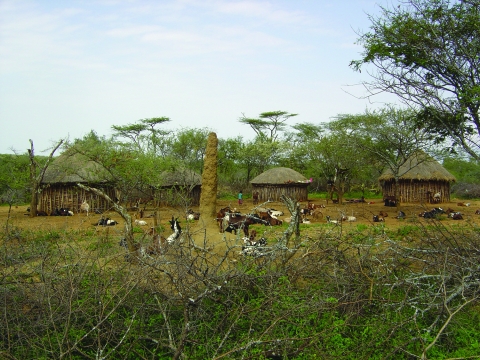
A large termite mound occupies the central area of this characteristic Pokot compound. The mound provides a resting and breeding site for the sandly vector of visceral leishmaniasis. Photographer: J.H. Kolaczinski.

MSF’s treatment of kala-azar is crucial because it reduces the human reservoir and hence transmission. However, current control activities only reach the tip of the iceberg: a large, underlying pool of infected and infectious persons likely exists (*8*,*9*).

Kala-azar in Uganda will not likely be controlled unless the epidemiology of the disease is better understood and preventive activities are undertaken. This knowledge gap is being addressed by a partnership among the Malaria Consortium, MSF, the London School of Hygiene and Tropical Medicine, and the Vector Control Division of the Ugandan Ministry of Health. A case-control study to determine the local risk factors of kala-azar is almost completed and will be followed by seroprevalence studies in several Pokot villages, using a similar approach to recent work in the Baringo District, Kenya (*9*). The results will be used to formulate an integrated control strategy aimed at achieving our ultimate goal of eliminating kala-azar from Uganda.

## Supplementary Material

Appendix Figure Map showing Pokot Country (shaded box) in eastern Uganda and western Kenya.
