# Supplementary figures and images for: Kala-azar Control, Uganda
Source: Emerg Infect Dis. 2007 Mar;13(3):507–9. doi: 10.3201/eid1303.060706 (PMC2725906; doi:10.3201/eid1303.060706)

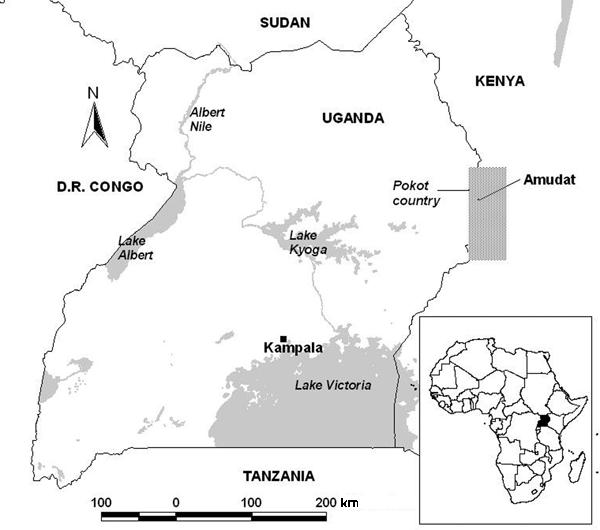

Supplement: Appendix Figure — Map showing Pokot Country (shaded box) in eastern Uganda and western Kenya. [file 06-0706_app-s1.gif]
